# Energy Balance Approach to Study the Role of Perspiration in Heat Distribution of Human Skin

**DOI:** 10.1155/2020/3154908

**Published:** 2020-03-09

**Authors:** Aijaz Mir, Ibrahim M. Almanjahie, Javid Gani Dar

**Affiliations:** ^1^Department of Mathematics, Government Degree College Kilam, Higher Education, Jammu and Kashmir, India; ^2^Department of Mathematics, College of Science, King Khalid University, Abha, Saudi Arabia; ^3^Statistical Research and Studies Support Unit, King Khalid University, Abha, Saudi Arabia; ^4^Department of Mathematical Sciences, Islamic University of Science and Technology, Jammu and Kashmir, India

## Abstract

This paper develops a model to identify the role of perspiration in temperature distribution of human skin. The model has been solved by using the energy balance equation on the surface of human skin. The role played by thermal conductance, convection, and heat radiation during heat transfer in human skin has been considered, and the relevant laws such as Fourier law for conduction, Newton's Law for convection, and Stefan–Boltzmann's law for radiation have been used in the model. Pennes' bioheat equation has been employed to estimate the heat flow in the dermal region of skin including subcutaneous tissue.

## 1. Introduction

Human beings have the ability to maintain nearly a constant core temperature of *T*_*c*_=37°C under a wide range of environmental conductions by controlling their rate of heat production and heat loss [1]. This process is called thermoregulation. The heat transfer with external environment takes place through conductance, convection, and radiation. The skin (including subcutaneous tissue) is the main organ which helps in thermoregulation of human body through conservation or dissipation of heat. Therefore, it becomes imperative to study the role of different parameters of the human skin involved in thermoregulation and the corresponding change in temperature distribution. Furthermore, as we know, in case of conduction of electric charges, there is a concept of electrical resistance, a similar analogy exists in heat transfer of human tissue, known as thermal resistance of the tissue, which is an important factor responsible for the control of heat loss and heat gain in extreme environmental conditions. In this paper, we shall discuss only heat transfer and the variation in peripheral temperature of human skin with respect to the various ambient temperatures. The concept of thermal resistance is left as a scope for the improvement of this paper.

Various researchers, for example Makrariya and Adlakha [2] and Xu et al. [3] formulated appropriate mathematical models to study the temperature distribution in human skin and subcutaneous tissue at high and low atmospheric temperatures. Makrariya and Adlakha [2] formulated a model for temperature distribution in dermal tissues of extended spherical organs of human body and solved it by the two-dimensional finite element method. Xu et al. [3] reviewed the theoretical and experimental study of heat transfer through skin tissue. Nadel et al. [4] conducted a study on the importance of skin temperature in the regulation of sweating and concluded that, at a constant core temperature, sweating rate is proportional to skin temperature, and at a constant temperature, sweating rate is proportional to the core temperature.

Earlier, Khanday et al. [5] estimated the effects of thermal stress on the behaviour of fluid concentrations in human dermal regions by formulating a mathematical model based on the diffusion equation along with appropriate boundary conditions and solving the model by the variational finite element method. Mir Aijaz et al. [6] used the variational finite element approach to study the thermal stress in multilayered human head.

In this paper, we shall assume balance of energy at the skin surface and obtain temperature profiles on the skin surface with respect to various environmental temperatures. The temperature distribution in the inner regions of human skin is calculated by Pennes bioheat equation [7]. Due to the convection property of heat flow, the fluid concentration of human tissue affects its heat transfer. Since there is a variation in the fluid concentration of human skin by perspiration, it becomes imperative to study the role of perspiration in heat transfer of human skin and subcutaneous tissue.

## 2. Assumptions of the Model

For the simplicity of solution and calculations, we adopt some assumptions of parameters used in the model. These assumptions are based on the fact that the role of these parameters is less significant in the resultant output of solved model as discussed by Seetharamu and Seetharamu [8]. These assumptions are as follows:

- Transfer of heat takes place in forward and backward directions only, i.e., the transfer of heat in the left and right direction is neglected.
- Although the parameters have different values in the different regions of human skin, all the parameters throughout a particular skin layer (epidermis, dermis, or subcutaneous tissue) are treated as uniform.
- A small surface of skin is under study, and the same is exposed to external environment for exchange of heat through radiation.
- Fluid is opaque to thermal radiation, and hence heat loss due to radiation in underlying layers of skin is neglected.

## 3. Mathematical Formulation

The outer surface of human skin is exposed to the environment, and its inner layer is at a temperature slightly less than the body core temperature which is approximately 37°C. In view of the calculation of heat transfer between human skin and its surroundings, it becomes optimum to focus on the heat distribution in various layers of skin. Let *ℓ* denote the thickness of skin, *T*_sf_ denote the temperature at the outer skin surface, *T*_*i*_ denote the temperature at the inner skin surface, and *T*_sd_ denote the temperature of the surrounding closed surface.

The transfer of heat in human skin mainly takes place by conduction, convection, and radiation. Through conduction, the heat flux *q*^″^(*W*/*m*^2^) is given by Fourier Law as

(1) $$\begin{array}{c} q^{\prime \prime }=-k\frac{\Delta T}{\Delta x}=-k\frac{T_{i}-T_{\text{sf}}}{\ell }, \end{array}$$

where *k* is the thermal conductivity. For convection, using Newton's law of cooling and incorporating the role of evaporation and latent heat, the heat flux *q*^″^(*W*/*m*^2^) is given by

(2) $$\begin{array}{c} q^{\prime \prime }=h\Delta T+\text{LE}, \end{array}$$

where *h* is the heat transfer coefficient, L is the late heat, and E is the evaporation rate. Through radiation, the heat flux *q*^″^(*W*/*m*^2^) is given by Stefan–Boltzmann Law as

(3) $$\begin{array}{c} q^{\prime \prime }=\varepsilon \sigma \left(T_{\text{sf}}^{4}-T_{\text{sd}}^{4}\right), \end{array}$$

where *ε* denotes the emissivity and is approximately equal to the absorption and *σ* is the Stefan–Boltzmann constant.

To deal with the complex structure of the human body, different methods have been used for heat transfer by many researchers. Aijaz et al. [6, 9] used the variational finite element method for the numerical solutions of the bioheat equation and justified their claim that this method gives optimum results for irregular geometrical patterns. In the present case, we shall use the surface energy balance method to determine the heat transfer. We apply the conservation of energy requirement at the surface of the medium. In this method, the controlled surfaces are located on either side of the boundary and enclose no mass or volume. Accordingly, the generation and storage terms of the conservation expression are no longer relevant, and it is only necessary to deal with surface phenomena. For this case, the conservation requirement becomes

(4) $$\begin{array}{c} {\dot{E}}_{\text{in}}-{\dot{E}}_{\text{out}}=0. \end{array}$$

## 4. Solution of the Model and Numerical Computation

The temperature profiles of the various layers of skin may be calculated by assuming an energy balance at the skin surface. That is,

(5) $$\begin{array}{c} {\dot{E}}_{\text{in}}-{\dot{E}}_{\text{out}}=0\\ \text{or }{\dot{E}}_{\text{in}}={\dot{E}}_{\text{out}}\\ \text{or }k\left(\frac{T_{i}-T_{\text{sf}}}{\ell }\right)=h\left(T_{\text{sf}}-T_{\text{sd}}\right)+\varepsilon \sigma \left(T_{\text{sf}}^{4}-T_{\text{sd}}^{4}\right)+\text{LE}. \end{array}$$

The only unknown variable is *T*_sf_, but we cannot solve it explicitly because of the fourth power dependence of the radiation term. Therefore, the approximate solution can be obtained by rewriting the above equation as

(6) $$\begin{array}{c} k\frac{T_{i}-T_{\text{sf}}}{\ell }=h\left(T_{\text{sf}}-T_{\text{sd}}\right)+h^{\prime }\left(T_{\text{sf}}-T_{\text{sd}}\right)+\text{LE}\\ \text{or }k\frac{T_{i}-T_{\text{sf}}}{\ell }=\left(h+h^{\prime }\right)\left(T_{\text{sf}}-T_{\text{sd}}\right)+\text{LE}. \end{array}$$

Solving *T*_sf_, we get

(7) $$\begin{array}{c} T_{\text{sf}}=\frac{\left(kT_{i}|/|\ell \right)+\left(h+h^{\prime }\right)T_{\text{sd}}-\text{LE}}{\left(k/\ell \right)+\left(h+h^{\prime }\right)}, \end{array}$$

where *h*′=*εσ*(*T*_sf_+*T*_sd_)(*T*_sf_^2^+*T*_sd_^2^), and its value is obtained by the assumption *T*_sf_ ~ *T*_sd_. Then, by using the value of *h*′ in Equation (7), we calculate the value of *T*_sf_. To estimate the temperature profiles of epidermis, dermis, and subcutaneous tissue, we have to neglect the heat transfer through radiation and incorporate role of the metabolic heat generation, and therefore it becomes imperative to use Pennes' bioheat equation [7] which is most appropriate as discussed by Myers [10] and Aijaz and Javid [11].

The governing equation, earlier used by Perl [12], for the heat flow in human tissue is given by

(8) $$\begin{array}{c} \rho c\frac{\partial T}{\partial t}=\text{div}\left(k\text{ grad }T\right)+m_{b}c_{b}\left(T_{A}-T\right)+S, \end{array}$$

where *T* is the temperature, *c* is the specific heat of tissue, *ρ* is the density of the tissue, *k* is the thermal conductivity of the system, *S* is the rate of metabolic heat generation, *c*_*b*_ is the specific heat capacity of the blood, *T*_*A*_ is arterial temperature, and *m*_*b*_ is the blood mass flow rate. Although there are various methods to solve equation (8), for example, see Khanday et al. [13, 14], it can be easily solved for the steady state case, and consequently equation (8) reduces to

(9) $$\begin{array}{c} \left(D^{2}-A^{2}\right)T=B, \end{array}$$

where *D* = *d*/d*x*, *A*^2^ = *m*_*b*_*c*_*b*_/*k*, and *B* = −((*m*_*b*_*c*_*b*_*T*_*A*_ + *S*)/*k*)

The complete solution of Equation (9) is

(10) $$\begin{array}{c} T=C_{1}e^{Ax}+C_{2}e^{-Ax}-\frac{B}{A^{2}}. \end{array}$$

The human skin is exposed to different environmental conditions with the skin surface temperature as *T*_sf_, and the inner temperature of subcutaneous tissue is *T*_0_. Therefore, the boundary conditions are taken as (a) *T* = *T*_sf_ at *x* = 0 and (b) *T* = *T*_0_ at *x* = *ℓ*

Using the boundary conditions (a) and (b) in Equation (10), the expression for the arbitrary constants *C*_1_ and *C*_2_ is given by *C*_1_ = (*α* − *βe*^−*Aℓ*^)/*A*^2^(*e*^*Aℓ*^ − *e*^−*Aℓ*^) and. *C*_2_ = (*α* − *βe*^*Aℓ*^)/*A*^2^(*e*^*Aℓ*^ − *e*^−*Aℓ*^), where *α* = *A*^2^*T*_0_ + *B* and *β* = *A*^2^*T*_sf_ + *B*

Now, using the values of the arbitrary constant in Equation (10), we get

(11) $$\begin{array}{c} T=\frac{\left[\alpha -\beta e^{-A\ell }\right]e^{Ax}-\left[\alpha -\beta e^{-A\ell }\right]e^{-Ax}-B\left(e^{A\ell }-e^{-A\ell }\right)}{A^{2}\left(e^{A\ell }-e^{-A\ell }\right)}. \end{array}$$

**Figure 1:**
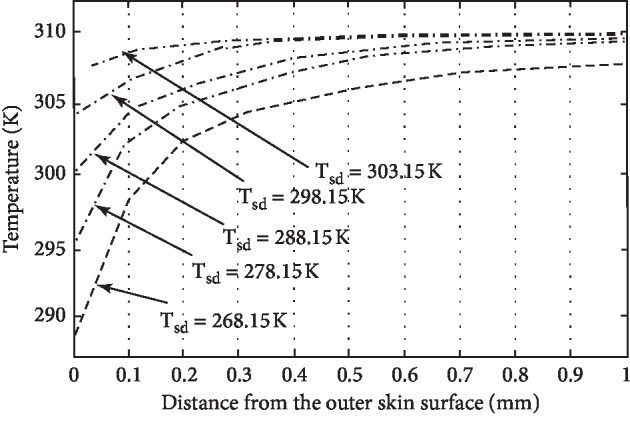
Temperature distribution of human skin at external temperature *T*_sd_ ≤ 303.15*K* and ignoring the role of perspiration.

**Figure 2:**
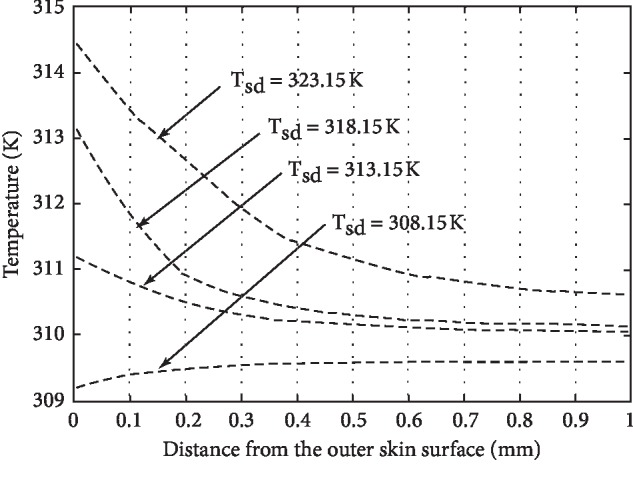
Temperature distribution of human skin at external temperature *T*_sd_ ≥ 308.15*K* and ignoring the role of perspiration.

**Figure 3:**
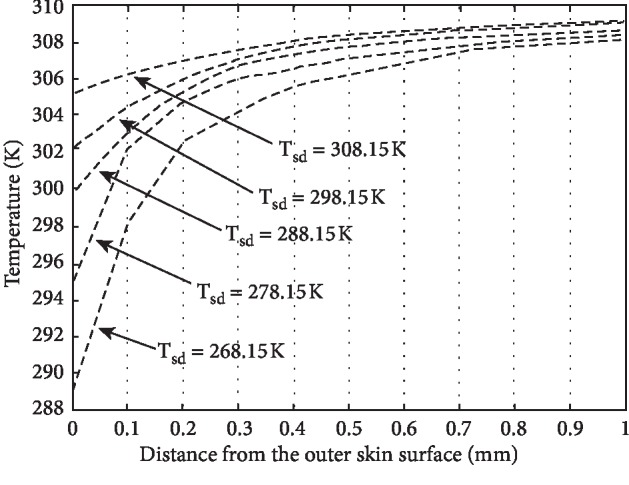
Temperature distribution of human skin at external temperature *T*_sd_ ≤ 303.15*K* and incorporating the role of perspiration.

**Figure 4:**
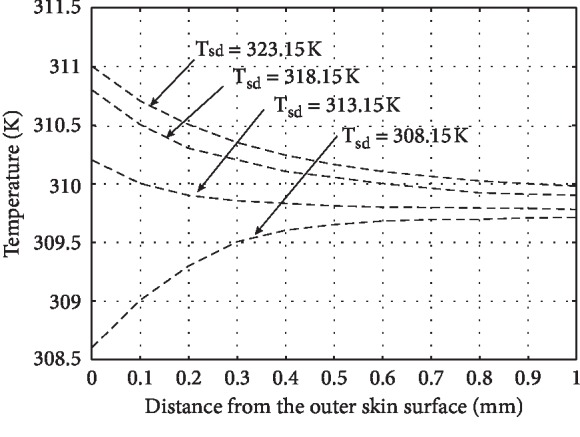
Temperature distribution of human skin at external temperature *T*_sd_ ≥ 308.15*K* and incorporating the role of perspiration.

For the numerical solution to the model graphical representation of results, it becomes optimistic to realize the role of different parameters used in the model. The different parameters effecting the temperature distribution of human skin are physiology of the individual and his size, weight, colour, and ability to adopt the abnormal environment. The environmental parameters effecting the temperature distribution are humidity, wind, and distance from equator or sea. In this paper, the role of all those parameters having significant role in the process of heat distribution in human tissue have been incorporated, and assumptions were applied for the parameters having less significance, but the role of perspiration has been noticed and displayed graphically as shown in Figures 1–4. If *H*_*v*_ denotes the heat required to evaporate one gram of water from human body, then the relation

(12) $$\begin{array}{c} Q=H_{v}m \end{array}$$

gives us the amount of heat *Q* required to produce *m* grams of perspiration. Consequently, the relation

(13) $$\begin{array}{c} \Delta T=\frac{Q}{\text{mc}}, \end{array}$$

where *c* is specific heat of skin, will give the corresponding temperature change on the skin surface.

For the numerical estimation of *T*_sf_ and *T* from equations (7) and (10), respectively, the numerical values of the parameters used in the model are needed.  Table 1 gives the suitable numerical approximation of these parameters.

## 5. Discussion and Conclusion

The main aim of this paper is to study the temperature distribution in human skin and subcutaneous tissue at different environmental temperatures. The temperature profiles have been obtained by using energy balance equation after keeping the energy balance at the skin surface. The different parameters such as specific heat, thermal conductivity, and rate of metabolic heat generation having their role in temperature distribution in the human body have been considered and two special cases *viz.*, (*i*) presence and (*ii*) absence of perspiration have been studied at both high and low temperatures.  Figure 1(*T*_sd_ ≤ 303.15*K*) and Figure 2(*T*_sd_ ≥ 308.15*K*) show the temperature distribution in the absence of perspiration at the temperatures (in Kelvin) as indicated against each curve. Similarly, Figure 3(*T*_sd_ ≤ 303.15*K*) and Figure 4(*T*_sd_ ≥ 308.15*K*) show the temperature distribution in presence of perspiration at the temperatures (in Kelvin) as indicated against each curve. The relation between the temperature distribution in skin and perspiration is very important for the maintenance of bearable temperature in skin and subcutaneous tissue, and moreover, there is a direct relation between thermal properties and burn injury as discussed by Jiang et al. [15], Ng and Chua [16], Wang and Qin [17], Aijaz and Khanday [18], and Xu et al. [19]. Therefore, perspiration plays an important role in reducing the chances of thermal injury. At high temperatures, the perspiration increases the production of sweat on skin surfaces, making it wet, thereby saving the skin form burn injury.

By entering the energy balance and appropriate input parameters in Equation (7), a model of the system may be developed for calculating *T*_sf_, and similarly the rate of heat loss at skin surface can be found from *q*_sf_=*KA*((*T*_*i*_ − *T*_*sf*_)/*ℓ*). With this model, parameter sensitivity studies may be performed to explore, for example, the effect of changing *h* and *T*_sf_.

This model is very important for studying the behaviour of human skin when exposed to abnormal temperatures especially during medical treatments as demonstrated by Khanday et al. [20] and Pearce and Thomsen [21]. The ability to design many medical devices and to develop the appropriate protocol for their use hinges on the engineer's ability to predict and control the distribution of temperature during thermal treatment and the distribution of the chemical species in chemotherapies. The treatment of mammalian tissue is made complicated by the morphology of this tissue. The blood flow within the venular and capillary structure of thermally-treated area affects heat transfer through advection processes. Later, veins and arteries, which commonly exist in pairs throughout the body, carry blood at different temperatures and advect thermal energy at different rates. Therefore, the veins and arteries exist in counterflow heat exchange arrangement with warm, arteriolar blood exchanging thermal energy with the cooler, venular blood through the intervening solid tissue. Networks of smaller capillaries can also affect local temperatures by perfusing blood through the treated area.

## Figures and Tables

**Table 1:** Physiological and numerical values of the parameters.

Quantity	Value	Units	Quantity	Value	Units
*k*_1_ (epi.)	0.31	*W*/*mK*	*S*_1_ (Epi.)	0	*W*/*m*^2^
*k*_2_ (der.)	0.29	—	*S*_2_ (der.)	6.278 × 10^2^	–
*k*_3_ (sub)	0.25	—	*S*_3_ (Sub.)	3.847 × 10^3^	–
*c*_1_ (epi.)	2.3 × 10^3^	*W*/*kg*/*K*	*L*	2.42	*J* · *g*^−1^
*c*_2_ (der.)	2.2 × 10^3^	—	*E*	8.33 × 10^3^	*l* · *m*^−2^ · *h*^−1^
*c*_3_ (sub.)	3.2 × 10^3^	—	*H*_*v*_	0.33	*J*/*kg*
*h*	2	*W*/*m*^2^ *K*	*ε*	0.6	−*c*
*σ*	5.670 × 10^−8^	*Wm* ^−2^ *K* ^−4^			

## Data Availability

The simulated data used to support the findings of this study are included within the article.
